# Cranial Neuropathies Associated With Pediatric Brain Tumors: Evaluation of Pediatric Otolaryngology Referral Patterns and Interventions

**DOI:** 10.7759/cureus.110628

**Published:** 2026-06-10

**Authors:** Ebone E Bady, Joely R Gendler, Fadlullah Ba'th, Sivakumar Chinnadurai, Brianne B Roby

**Affiliations:** 1 Department of Otolaryngology - Head and Neck Surgery, University of Minnesota, Minneapolis, USA; 2 Department of Otolaryngology, University of Minnesota School of Medicine, Minneapolis, USA; 3 Department of Pediatric Otolaryngology and Facial Plastic Surgery, Children's Hospitals and Clinics of Minnesota, Minneapolis, USA

**Keywords:** cranial nerve deficits, cranial neuropathy, facial paralysis, otolaryngology referrals, pediatric cns tumor

## Abstract

Background and aim

Otolaryngologists play a crucial role in managing cranial nerve (CN) deficits in pediatric brain tumors. This study aims to determine rates of persistent cranial neuropathy and associated otolaryngology referral.

Methods

A total of 109 pediatric patients with primary intracranial neoplasms and documented CN deficits were retrospectively identified. Outcomes of interest included CN deficit, otolaryngology referral, recovery status, and surgical interventions. Variables were analyzed using chi-square and Fisher’s exact tests.

Results

Cranial neuropathies persisted in 69 (63.3%) patients, the majority with posterior fossa tumors. Common deficits included facial weakness (N = 62, 56.9%), ophthalmoplegia (N = 59, 54.1%), vocal fold paresis (N = 11, 10.1%), dysphagia (N = 11, 10.1%), hearing loss (N = 9, 8.3%), and trigeminal neuropathy (N = 7, 6.4%). Twenty-two (20.2%) patients were referred to otolaryngology. Referred patients were significantly less likely to have achieved full recovery (X² = 5.53, p = 0.02) and more likely to undergo corrective intervention (X² = 15.88, p < 0.01). Vocal fold paresis, dysphagia, and hearing loss were each associated with referral (p < 0.01), whereas facial neuropathy, ophthalmoplegia, and trigeminal neuropathy were not. Notably, 51 of 87 (58.6%) non-referred patients demonstrated no recovery of their cranial neuropathy.

Conclusions

Despite high rates of persistent cranial neuropathies among pediatric patients with intracranial neoplasms, otolaryngology referrals remain notably infrequent, particularly for facial paralysis. Earlier and more systematic referrals may represent a meaningful opportunity to improve functional outcomes through earlier and timely intervention.

## Introduction

Pediatric brain tumors are the second most common childhood cancers and are the leading cause of cancer-related mortality in children younger than 20 years of age [1]. Between 2016 and 2020, the incidence of pediatric brain tumors was 6.13 per 100,000 [2]. Common presenting symptoms associated with pediatric brain tumors include headache, vomiting, visual changes, and cranial nerve (CN) injury. Cranial neuropathies commonly persist after treatment and may become increasingly prominent over time [3]. Factors contributing to more significant neurologic deficits include tumor location, history of prior craniotomy, and the presence of neurologic deficits prior to treatment [4]. In the pediatric population, tumors of the posterior fossa (PF) account for nearly half of all pediatric CNS tumors [5]. PF tumors pose a particular risk for postoperative neuropathies due to their proximity to critical neurovascular structures, including multiple CN nuclei. Among pediatric patients with PF tumors, one-third will experience lower CN deficits [6].

Otolaryngologists play a crucial role in evaluating CN deficits and are generally consulted to assess facial asymmetry, hoarseness, dysphagia, respiratory protection, hearing loss, and imbalance. To date, no studies have systematically examined referral patterns to otolaryngologists among pediatric patients with cranial neuropathies attributable to their tumor or its treatment. In order to better characterize otolaryngology referral patterns, neurological sequelae, and the need for postoperative intervention in pediatric patients treated for brain tumors, this study reviews the oncology database from a single tertiary pediatric hospital. We aim to determine the rate of referral to otolaryngology and the need for intervention for cranial neuropathies following treatment for pediatric brain tumors. We hypothesize that rates of pediatric otolaryngology referral would be higher among patients with persistent CN deficits.

## Materials and methods

Following Institutional Review Board approval, all pediatric patients aged 0-18 years who underwent treatment for a brain tumor between December 2000 and December 2015 at a tertiary pediatric hospital were included in this study. Patients were included based on the following ICD codes with corresponding records in the medical chart: ICD-9-CM 350-352 and ICD-10-CM G52.0-G52.3, G52.8. Charts were retrospectively reviewed for documented CN deficits in providers’ notes beginning at the time of primary intracranial neoplasm diagnosis through the most recent clinical visit (until 2015). Inclusion criteria required that patients be younger than 18 years of age at the time of CN deficit onset and that there be evidence of a deficit after treatment of the intracranial neoplasm. Exclusion criteria included the absence of documented cranial neuropathy and no receipt of surgical, radiation, or chemotherapy treatment at our institution. Referrals to otolaryngology were identified and recorded. If applicable, surgical interventions for cranial neuropathies were documented. Recovery of cranial neuropathy was categorized as full recovery, partial recovery, or no recovery based on the most recent clinical visit prior to interventional treatment or otolaryngology consultation, if applicable. Full recovery was defined as spontaneous resolution of neuropathy without intervention. Partial recovery was defined as improved but incomplete resolution of neuropathy. No recovery was defined as persistence of cranial neuropathy without demonstrable improvement. Additional data regarding tumor type, grade, site as defined by surgical pathology/histology, and treatment modality for primary tumor (i.e., surgery, radiation, and/or chemotherapy) were also obtained.

Comparisons of categorical data were performed using two-tailed chi-square goodness-of-fit and Fisher’s exact tests. Statistical significance was set at an α level of 0.05. All statistical analyses were performed using R version 4.1.2 (R Foundation for Statistical Computing, Vienna, Austria).

## Results

Cohort

Between 2000 and 2015, 109 new cases of primary intracranial neoplasms with recorded CN deficits were diagnosed. The PF region was the most common tumor site, accounting for 79 (72.5%) cases. Patients seen by pediatric otolaryngologists had higher rates of PF tumors. A detailed breakdown of tumor characteristics, location, and treatment can be found in Table 1.

**Table 1:** Tumor characteristics, location, and treatment of primary intracranial neoplasms in pediatric patients with cranial neuropathy based on otolaryngology evaluation status Percentages were calculated using the total cohort count within each column. C = chemotherapy; PF = posterior cranial fossa; R = radiation; S = surgery

Tumor characteristics	Whole cohort (n = 109)	Seen by otolaryngology (n = 22)	Not seen by otolaryngology (n = 87)
Location, N (%)			
PF	79 (72.5%)	19 (86.4%)	60 (69.0%)
Other	30 (27.5%)	3 (13.6%)	27 (31.0%)
Tumor pathology, N (%)			
Astrocytoma	35 (32.1%)	3 (13.6%)	32 (36.8%)
Medulloblastoma	31 (28.4%)	8 (36.4%)	23 (26.4%)
Ependymoma	12 (11.0%)	5 (22.7%)	7 (8.1%)
Atypical teratoid rhabdoid tumor	3 (2.8%)	0 (0.0%)	3 (3.5%)
Schwannoma	3 (2.8%)	2 (9.1%)	1 (1.1%)
Glioma	5 (4.6%)	0 (0.0%)	5 (5.7%)
Craniopharyngioma	6 (5.5%)	0 (0.0%)	6 (6.9%)
Pleomorphic xanthoastrocytoma	2 (1.8%)	0 (0.0%)	2 (2.3%)
Other	12 (11.0%)	4 (18.2%)	8 (9.2%)
Tumor treatment, N (%)			
S	49 (44.9%)	3 (13.6%)	46 (52.9%)
C	4 (3.7%)	2 (9.1%)	2 (2.3%)
R	0 (0.0%)	0 (0.0%)	0 (0.0%)
S + R	9 (8.3%)	3 (13.6%)	6 (6.9%)
S + C	13 (11.9%)	2 (9.1%)	11 (12.6%)
R + C	3 (2.8%)	2 (9.1%)	1 (1.2%)
S + C + R	31 (28.4%)	10 (45.5%)	21 (24.1%)

Greater than one cranial neuropathy was identified in 34 (31.2%) patients. Of the 109 cases with CN deficits, 69 (63.3%) demonstrated no recovery, 29 (26.6%) achieved full recovery, and 11 (10.1%) exhibited partial recovery. Recovery status did not differ by treatment modality (e.g., surgery, chemotherapy, and/or radiation; Fisher’s exact test, p > 0.05). Of the 109 patients, 62 (56.9%) presented with facial palsy/weakness, 59 (54.1%) with ophthalmoplegia, 11 (10.1%) with vocal fold paresis, 11 (10.1%) experienced dysphagia, nine (8.3%) with hearing loss, and seven (6.4%) with trigeminal neuropathy (Table 2).

**Table 2:** CN deficits and rates of recovery in pediatric patients with primary intracranial neoplasms based on otolaryngologist evaluation status ^*^ Statistically significant differences between otolaryngology referral (or no referral) with p-values < 0.05. ^†^ Patients who were referred to otolaryngology were significantly less likely to have a full recovery (compared with none or partial recovery). Percentages were calculated using the total cohort count within each column. CN = cranial nerve

CN deficit	Whole cohort (n = 109)	Seen by otolaryngology (n = 22)	Not seen by otolaryngology (n = 87)	p-value
>1 CN deficit, N (%)	34 (31.2%)	11 (50.0%)	23 (26.4%)	0.06
CN deficit, N (%)				
Facial palsy	62 (56.9%)	13 (59.1%)	49 (56.3%)	1
Ophthalmoplegia	59 (54.1%)	8 (36.4%)	51 (58.6%)	0.1
Vocal cord paresis	11 (10.1%)	8 (36.4%)	3 (3.4%)	p < 0.01^*^
Swallowing dysfunction	11 (10.1%)	6 (27.3%)	5 (5.7%)	p < 0.01^*^
Hearing loss	9 (8.3%)	7 (31.8%)	2 (2.3%)	p < 0.01^*^
Trigeminal neuropathy	7 (6.4%)	3 (13.6%)	4 (4.6%)	0.14
Recovery of CN deficit, N (%)				
None	69 (63.3%)	18 (81.8%)	51 (58.6%)	-
Full	29 (26.6%)	1 (4.5%)	28 (32.2%)	0.02^*†^
Partial	11 (10.1%)	3 (13.6%)	8 (9.2%)	-
Intervention for CN deficit, N (%)	20 (18.3%)	11 (50.0%)	9 (10.3%)	p < 0.01^*^

Patients not referred to otolaryngology

A pediatric otolaryngologist did not evaluate 87 (79.8%) patients. Among these patients, 51 (58.6%) demonstrated no recovery, 28 (32.2%) achieved full recovery, and eight (9.2%) exhibited partial recovery (Table 2). Nine (10.3%) patients received corrective interventions outside of otolaryngology, all of whom had either no recovery or partial recovery. Fifteen patients died during the follow-up period.

Patients referred to otolaryngology

Twenty-two (20.2%) of the total 109 patients were referred to otolaryngology for further evaluation. Among these patients, 18 (81.8%) had no recovery, one (4.5%) had full recovery, and three (13.6%) experienced partial recovery upon consultation. Patients who were seen by an otolaryngologist were significantly less likely (X²(1) = 5.53, p = 0.02) to have achieved full recovery. Eleven (50.0%) presented with more than one cranial neuropathy (Table 2).

Patients evaluated by otolaryngology were significantly more likely to undergo a procedure (X²(1) = 15.88, p < 0.01). Surgical interventions were performed by otolaryngologists in 11 (50.0%) of the 22 patients. A total of 14 procedures were performed on these 11 patients, as some patients underwent more than one intervention. Procedures included facial nerve reanimation (N = 5), injection laryngoplasty (N = 3), tracheostomy (N = 2), eyelid weight placement (N = 1), palatal adhesion (N = 1), pharyngeal flap (N = 1), and recurrent laryngeal nerve reanastomosis (N = 1). None of the four patients who demonstrated at least partial or full spontaneous recovery received an intervention after being evaluated by otolaryngology.

Nerve deficits

Of the 62 patients with facial palsy, 18 (29.0%) fully recovered. Thirteen (21.0%) of the 62 patients were referred to otolaryngology for further evaluation, with 12 of these cases demonstrating persistent deficit (N = 9) or partial recovery (N = 3). Among unreferred patients with CN VII deficits, 25 (51.0%) had no recovery, 17 (34.7%) spontaneously recovered, and seven (14.3%) partially recovered (Table 3). There was no statistically significant difference between facial nerve deficit and referral to otolaryngology for further evaluation (Fisher’s exact test, p > 0.05, Table 2).

**Table 3:** Rates of recovery within each CN deficit in pediatric patients with primary intracranial neoplasms based on otolaryngologist evaluation status Percentages for deficit frequency categorized by otolaryngology referral are based on the total cohort amount within each row (bolded). Percentages broken down by recovery status are based on the deficit column total within the referral status categorization (non-bold). CN = cranial nerve

CN deficit	Whole cohort (n = 109)	Seen by otolaryngology (n = 22)	Not seen by otolaryngology (n = 87)
Facial palsy (N, %)	62 (56.9%)	13 (21.0%)	49 (79.0%)
No recovery	34 (54.8%)	9 (69.2%)	25 (51.0%)
Fully recovered	18 (29.0%)	1 (7.7%)	17 (34.7%)
Partial recovery	10 (16.1%)	3 (23.1%)	7 (14.3%)
Ophthalmoplegia (N, %)	59 (54.1%)	8 (13.6%)	51 (86.4%)
No recovery	41 (69.5%)	7 (87.5%)	34 (66.7%)
Fully recovered	12 (20.3%)	0 (0.0%)	12 (23.5%)
Partial recovery	6 (10.2%)	1 (12.5%)	5 (9.8%)
Vocal cord paresis (N, %)	11 (10.1%)	8 (72.7%)	3 (27.3%)
No recovery	7 (63.6%)	5 (62.5%)	2 (66.7%)
Fully recovered	1 (9.1%)	0 (0.0%)	1 (33.3%)
Partial recovery	3 (27.3%)	3 (37.5%)	0 (0.0%)
Swallowing dysfunction (N, %)	11 (10.1%)	6 (54.5%)	5 (45.5%)
No recovery	9 (81.8%)	5 (83.3%)	4 (80.0%)
Fully recovered	0 (0.0%)	0 (0.0%)	0 (0.0%)
Partial recovery	2 (18.2%)	1 (16.7%)	1 (20.0%)
Hearing loss (N, %)	9 (8.3%)	7 (77.8%)	2 (22.2%)
No recovery	8 (88.9%)	6 (85.7%)	2 (100%)
Fully recovered	0 (0.0%)	0 (0.0%)	0 (0.0%)
Partial recovery	1 (11.1%)	1 (14.3%)	0 (0.0%)
Trigeminal neuropathy (N, %)	7 (6.4%)	3 (42.9%)	4 (57.1%)
No recovery	5 (71.4%)	3 (100.0%)	2 (50.0%)
Fully recovered	2 (28.6%)	0 (0.0%)	2 (50.0%)
Partial recovery	0 (0.0%)	0 (0.0%)	0 (0.0%)

Ten (90.9%) of the 11 patients with vocal cord paresis did not achieve full recovery (no recovery, N = 7; partial recovery, N = 3). Eight (72.7%) of these 11 patients were evaluated by otolaryngology for further evaluation, none having achieved full recovery (Table 3). Patients with vocal cord paresis were significantly more likely to receive otolaryngology referral (Fisher’s exact test, p < 0.01, Table 2) and significantly more likely to receive corrective treatment (X²(1) = 8.18, p < 0.01).

None of the patients with dysphagia achieved full spontaneous recovery, although two (18.2%) demonstrated partial recovery. Six (54.5%) of the total 11 patients were referred to otolaryngology (Table 3). There was a significant association between dysphagia and referral to otolaryngology (Fisher’s exact test, p < 0.01, Table 2). Patients with dysphagia were significantly more likely to receive corrective treatment (X²(1) = 4.16, p = 0.04). One unreferred patient with dysphagia required placement of a gastrojejunostomy feeding tube.

Of the nine patients with sensorineural hearing loss (SNHL), chemotherapy was utilized in six (66.7%). Hearing loss was noted post-radiation or post-surgical intervention, but before chemotherapy in two of these six patients. Nearly all hearing deficits persisted: eight (88.9%) demonstrated no recovery, and one (11.1%) showed partial recovery. Seven (77.8%) of the total patients were referred to otolaryngology (Table 3). Patients with SNHL were significantly more likely to be referred to otolaryngology compared with those without a deficit (Fisher’s exact test, p < 0.01, Table 2).

The distribution of recovery in ophthalmoplegia was as follows: 41 (69.5%) patients with no recovery, 12 (20.3%) with spontaneous recovery, and six (10.2%) with partial recovery (Table 3). Of the 59 patients with ophthalmoplegia, eight (13.6%) were referred to otolaryngology. Additionally, five (71.4%) cases of trigeminal neuropathy were persistent, with three of these persistent cases being referred to otolaryngology (Table 3). Neither ophthalmoplegia nor CN V deficits were associated with otolaryngology referral (Table 2).

## Discussion

This investigation offers the first retrospective study to examine referral patterns to otolaryngologists for common cranial neuropathies associated with pediatric brain tumors. We identified 109 patients with pediatric brain tumors who were diagnosed with a coexisting cranial neuropathy. Of these patients, the majority presented with PF tumors (72.5%) with associated CN VII palsy (56.9%). While only 29 (26.6%) of the total 109 patients achieved full recovery, only 22 (20.2%) were referred to otolaryngology. Even after excluding patients who demonstrated partial recovery of their associated neuropathy, this still left 51 of the total 109 patients (46.8%) with persistent cranial neuropathy but no otolaryngology evaluation for potential interventions that could lead to improvement of their cranial neuropathy.

There have been significant improvements in survival rates for pediatric brain tumors and surgical treatment efforts in recent years [7]. However, postoperative complications, including CN palsies, remain relatively common. Sønderkaer et al. [8] evaluated long-term neurological outcomes in patients with childhood brain tumors treated surgically and found that 56.5% of patients had persistent postoperative CN deficits at a median follow-up length of 10.7 years. Our study found similar results, with 63.3% of patients having persistent cranial neuropathies. However, only 20.2% of patients were referred to otolaryngology for further evaluation. As survival continues to improve, attention must shift toward long-term quality of life.

**Figure 1:**
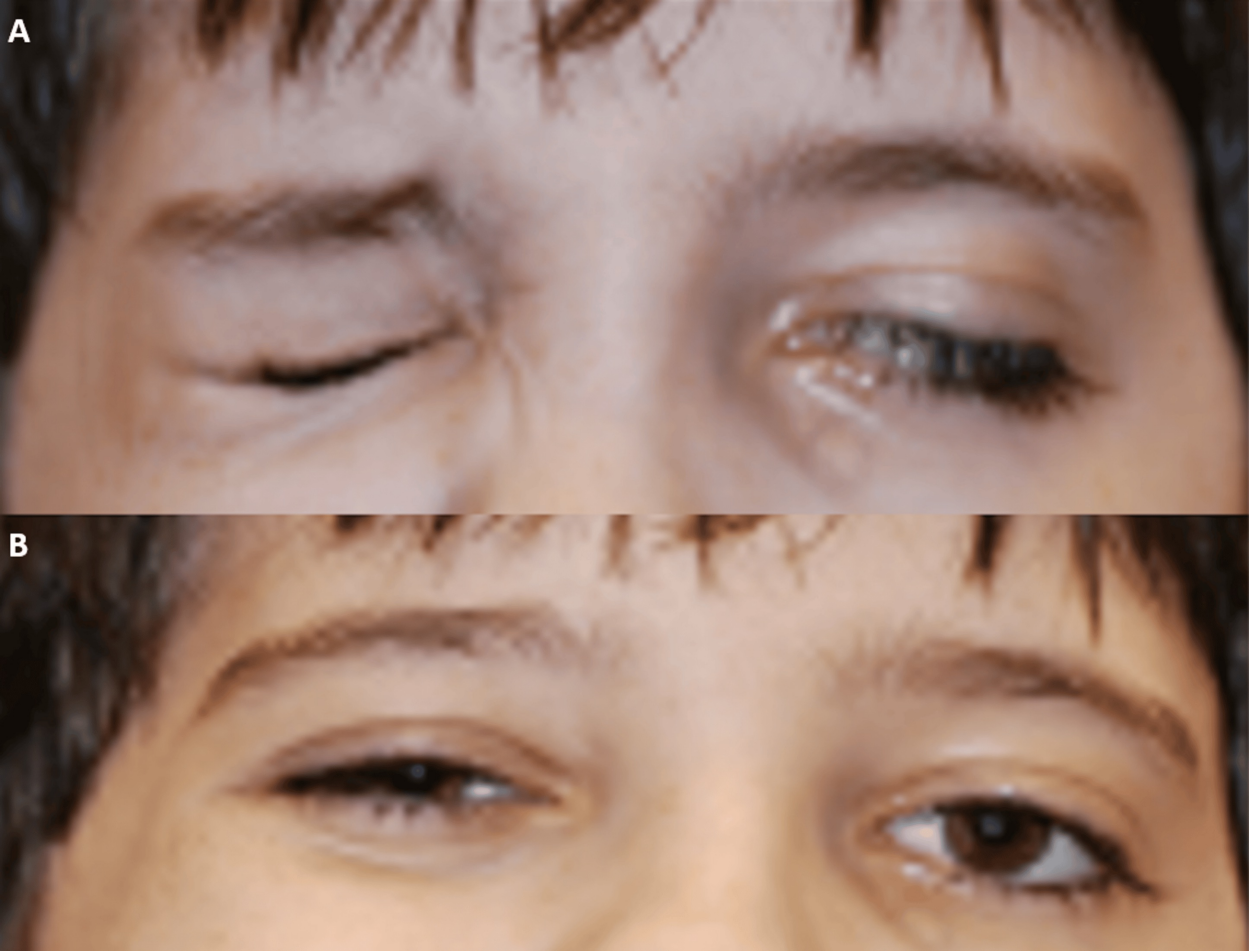
Facial nerve deficit related to pediatric brain tumor (A) Complete left-sided facial paralysis one year after surgical excision of a cerebellopontine angle tumor. (B) Left-sided eyelid weight placement to aid in closure.

CN deficits carry significant immediate and long-term aesthetic and functional consequences, particularly in developmental milestones. Facial weakness can result in drooling, corneal ulceration, hyperacusis, chewing deficits, etc. (Figure 1) [9]. Dysphagia as a consequence of pediatric brain tumor resection carries significant morbidity, including prolonged hospital stays, aspiration, malnutrition, delayed developmental milestones in children, and the potential need for inpatient rehabilitation [7,10]. Further, it can profoundly and negatively impact an individual’s quality of life [11]. Hearing deficits can adversely impact childhood learning experiences. With over half of the patients in this study demonstrating persistent CN deficits, it raises the question of how we can enhance functional and aesthetic well-being in this pediatric population.

This study did not find a significant association between treatment modality and recovery status. However, patients were significantly more likely to be seen by otolaryngology if they had no recovery and subsequently more likely to undergo an intervention for potential improvement once they were seen by otolaryngology. In other words, referrals may be deferred until deficits become sufficiently severe and persistent to the point where surgical correction becomes necessary.

In this study, 54.5% of patients with dysphagia, 72.7% of patients with vocal cord paresis, and 77.8% of patients with hearing deficits were referred to otolaryngology for consultation and treatment. All three deficits were significantly associated with otolaryngology referral (p < 0.01), indicating a well-established referral pattern for these particular cranial neuropathies. Moreover, patients with dysphagia and vocal cord paresis demonstrated a significantly higher likelihood of receiving corrective treatments for the associated deficit. It is encouraging to see such strong referral patterns coupled with appropriate treatment for these deficits, given their potential for life-threatening complications such as aspiration, airway compromise, and permanent hearing loss.

However, despite facial palsy being the most common cranial neuropathy in our cohort, it had one of the lowest rates of referral to otolaryngology, second only to ophthalmoplegia. Isolated ophthalmoplegias and cases severe enough to warrant intervention are more likely to have been referred to ophthalmology rather than otolaryngology, suggesting that some referrals may not have been captured and that overall referral rates for this CN deficit may even be underestimated. Still, only 21.0% of patients with facial palsy were referred to otolaryngology. This corresponds with a similar study in the United Kingdom analyzing referrals to pediatric otolaryngologists in children with CN VII paralysis; they reported that of the 172 general practitioners surveyed, only 22% referred to otolaryngologists for facial paralysis [12]. In our study, this left over half of the total patients with unresolved facial palsy receiving no otolaryngology referral. Collectively, these findings suggest the possibility of a referral bias regarding which cranial neuropathies are directed to otolaryngology.

While immediate intervention may not be necessary and spontaneous recovery from CN palsies (particularly CN VII) is known, early identification and evaluation of cranial neuropathies by a multidisciplinary team in children with brain tumors is best practice and promotes improved aesthetic and functional outcomes [13]. The interdisciplinary evaluation provides the clinical team with a comprehensive understanding of the varying phases of symptom evolution, thereby informing treatment or therapy options if or when they become necessary. This argues for immediate integration of otolaryngology into the multidisciplinary care paradigm for pediatric patients with brain tumors and CN deficits. Just as ophthalmology consultation is argued to be part of standard-of-care in pediatric CNS tumors due to involvement of the tumor or treatment plan on afferent and efferent visual systems [14], otolaryngology consultation should follow a similar line of reasoning to identify and manage other sensory complications or CN deficits.

Moreover, it is well established that various CN palsies can be effectively managed within the otolaryngology specialty. While treatment is tailored to the specific deficit, options may include surgical (i.e., facial reanimation, vocal cord injections, CN anastomosis, etc.), hearing aids, neuroactive medication, physical therapy, and/or speech-language pathology referral, etc. In fact, once patients in our cohort received a referral to otolaryngology, they were significantly more likely to receive a corrective treatment, demonstrating that these referrals fall squarely within the scope of otolaryngologic practice. One prior study examined dysphagia and dysphonia treatment outcomes in pediatric patients with neurologic malignancies and found that initiation of treatment (i.e., injection laryngoplasty) and therapy offered benefits/improvements in the majority of patients [15]. In our study, of the patients not evaluated by a pediatric otolaryngologist, 58.6% had no documented recovery, suggesting a potential opportunity for intervention. Our results highlight a gap in current referral patterns and a potential for enhancing patient outcomes.

Limitations

This study is limited by its relatively small sample size, particularly in the group referred to otolaryngology, which limits the statistical power of our analysis. Patients who underwent treatment near the end of the study period had the potential for unrecorded status changes (i.e., spontaneous recovery or future otolaryngology consultation), resulting in possible immortal time bias. Additionally, referrals to other specialists outside of otolaryngology (i.e., neurology, ophthalmology, etc.) were not recorded. Therefore, it is possible that patients with persistent cranial neuropathies were being evaluated for possible improvement, albeit outside of the care of otolaryngology. This may account for the relatively low referral rate reported in our study for patients with ophthalmoplegia, despite the majority demonstrating incomplete recovery. Moreover, six of the nine (66.7%) patients with hearing loss were treated with chemotherapy, and therefore, SNHL could be linked to chemotoxicity. However, two of these six patients exhibited hearing loss prior to initiation of chemotherapy, and three of the nine total patients had no chemotherapy as part of their treatment regimen. Those patients without chemotherapy-associated SNHL had tumors involving CN VIII or tumors compressing CN VIII and, therefore, can reliably be categorized as a cranial neuropathy.

This study did not systematically grade or distinguish CN deficit severity but instead assessed it based on presence, improvement, or absence using provider documentation. This introduces an aspect of heterogeneity within each referral status cohort. However, the primary objective of this study was to assess and quantify referral rates to otolaryngology in the presence of any persisting CN deficit, regardless of severity, as a preliminary means to systematically assess referral patterns. Future studies could consider using a grading system to better control and distinguish cohorts, as well as assess whether severity acts as a mediating factor for referral status. Future directions also include assessing referral timing and rehabilitation pathways for cranial neuropathies associated with pediatric brain tumors.

## Conclusions

CN palsies associated with pediatric brain tumors are common long-term post-treatment complications that can require targeted interventions. This study contributes to the scarce data to date that attempts to elucidate referral patterns to otolaryngologists after diagnosis of a pediatric brain tumor and associated cranial neuropathies commonly treated by otolaryngologists (facial weakness, hearing loss, dysphagia, and vocal cord paresis). From our cohort, the findings demonstrate that facial nerve paralysis is the most prevalent cranial neuropathy in PF tumors. In this study, most patients with CN VII paralysis were not referred to pediatric otolaryngology, making them less likely to have an intervention performed to aid in the correction of associated deficits. Untreated facial weakness, along with hearing loss, dysphagia, and vocal cord paresis, carries long-term sequelae that can limit a child’s educational progression and social interactions. Further studies are needed to understand referral patterns to specialists in pediatric brain tumor patients with CN palsies and the implications for patient outcomes with more comprehensive interdisciplinary evaluation and management. This data will ideally inform the development of a more streamlined, multidisciplinary approach to the management of pediatric cranial neuropathies arising from the treatment of brain tumors.
